# Circadian Clock, Time-Restricted Feeding and Reproduction

**DOI:** 10.3390/ijms21030831

**Published:** 2020-01-28

**Authors:** Xiaoyue Pan, Meredith J. Taylor, Emma Cohen, Nazeeh Hanna, Samantha Mota

**Affiliations:** 1 Department of Foundations of Medicine, New York University Long Island School of Medicine, Mineola, New York, NY 11501, USA; 2 Diabetes and Obesity Research Center, NYU Winthrop Hospital, Mineola, New York, NY 11501, USA; 3 Department of Pediatrics, NYU Winthrop Hospital, Mineola, New York, NY 11501, USA

**Keywords:** circadian clock, hormone regulation, time-restricted feeding, neural regulation, reproduction, reproductive failure

## Abstract

The goal of this review was to seek a better understanding of the function and differential expression of circadian clock genes during the reproductive process. Through a discussion of how the circadian clock is involved in these steps, the identification of new clinical targets for sleep disorder-related diseases, such as reproductive failure, will be elucidated. Here, we focus on recent research findings regarding circadian clock regulation within the reproductive system, shedding new light on circadian rhythm-related problems in women. Discussions on the roles that circadian clock plays in these reproductive processes will help identify new clinical targets for such sleep disorder-related diseases.

## 1. Introduction

**Figure 1:**
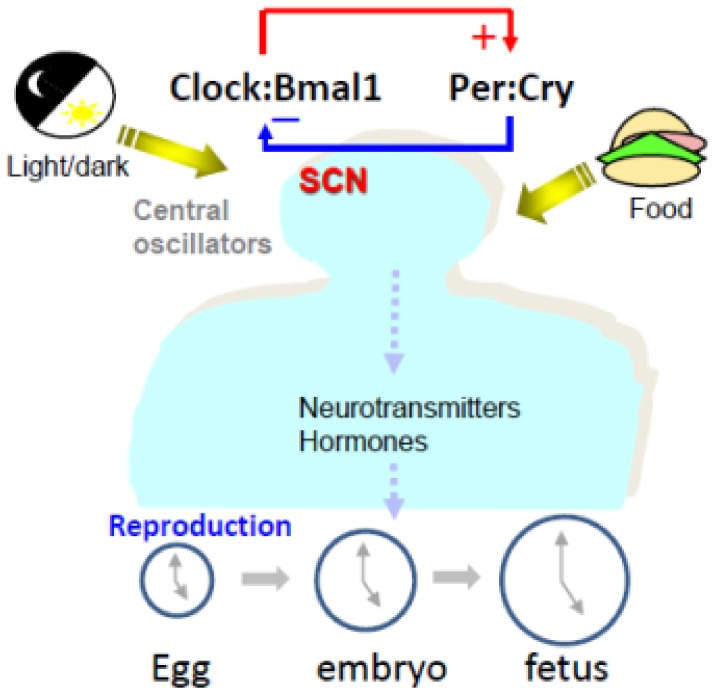
Light and Food intake are main input factors of circadian clock genes. In brain suprachiasmatic nucleus (SCN) as well as peripheral clock: Clock and Bmal1 heterodimerize to activate transcription of circadian target genes including the genes of Per 1/2/3 and Cry1/2). Per and Cry interact and inhibit Bmal1 and Clock. Clock genes rhythm are present in a wide array of other tissues (including the liver, kidney, lung, heart, etc.) and these tissues have their own rhythm distinct from that of the SCN. SCN circadian clock can control behavior, feeding and reproduction through neurotransmitters and hormones to regulate reproduction.

In mammals, the body’s clock is regulated through a series of various genes present in all organs such as *Period* genes (*Per 1/2/3,* Period Circadian Regulator1/2/3), *Cryptochrome* genes (*Cry 1/2*, Cryptochrome Circadian Regulator 1/2), Circadian Locomotor Output Cycles Kaput (*Clock*) gene, *and* Aryl hydrocarbon receptor nuclear translocator-like protein 1 (ARNTL, also known as MOP3 or Bmal1) gene [1,2,3,4,5,6,7]. In SCN as well as peripheral tissue, Clock and Bmal1 heterodimerize to activate transcription of circadian target genes, including the genes of *Per 1/2/3 and Cry1/2*)*. Per* and *Cry* interact and inhibit Bmal1 and Clock (Figure 1). The rhythmic expression of Clock genes rhythm is present in a wide array of tissues (including the liver, kidney, lung, and heart), and these tissues have their own rhythms distinct from that of the suprachiasmatic nucleus (SCN). SCN’s “circadian clock” genes can control behavior, feeding, and reproduction through neurotransmitters and hormones (Figure 1) [2,8]. These circadian clock genes drive the body’s circadian rhythm, and their disruption can lead to a host of issues such as cancer, obesity, and atherosclerosis [3,9,10,11,12,13,14,15,16]. Disruption of the clock genes has also been implicated in a variety of malfunctions of homeostasis, including glucose, and lipid metabolism [17,18,19,20]. Several circadian clock genes such as *Clock*, *Bmal1*, *Per2*, and *Cry1* are expressed in human and animal full-term placenta tissue, suggesting a potential circadian rhythm [21,22,23,24,25,26]. As demonstrated in prior research, the relationships between the placenta and fetal circadian signals are complex and are essential for the development of a successful pregnancy. Based on microarray studies of the liver, kidney, and heart tissues, it was revealed that some of the rhythms are driven directly by clock genes, whereas other aspects of these rhythms are induced by other tissue-specific transcription factors that are controlled by clock genes [24,27,28]. With the recent discovery that genes such as *Clock*, *Bmal1*, *Per2*, and *Cry1* are also present in the mammalian ovary, oviduct, uterus and placenta, observations have led to the hypothesis that disruption of the body’s natural clock has a negative effect on the embryo development and pregnancy [22,24]. Currently, however, there are few studies linking fertility problems to disruption in circadian clock genes in humans. 

**Figure 2:**
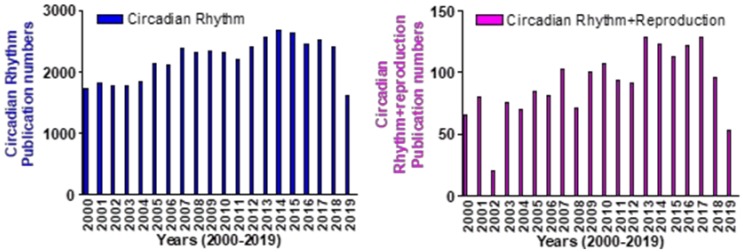
The number of references found for each year (From 2000 to 2019) of publication on the PubMed database using the keyword ‘Circadian Rhythm’ (left) or ‘Circadian Rhythm + Reproduction’ (right). In 2019, this number of Circadian Rhythm was 1621, the number of Circadian Rhythm reproduction was 53.

The SCN, located in the hypothalamus, is linked to optic cues. In addition to being responsible for the establishment of endogenous rhythms in mammals, the SCN also influences the circadian timing in individual cells. The reproductive control of various species is connected to circadian rhythm via light and the length of days. This control is linked to a series of feedback loops in the SCN. Many aspects of reproductive biology are regulated by the circadian rhythm [29,30]. This includes the estrus cycle, levels of luteinizing hormone (LH), ovulation, production and maturation of sperm, fertilization, insemination, and embryo implantation [23,31]. This internal clock can subtly regulate or be an integral part of the process of reproduction. It has been found that the disruption of certain primary genes such as *Clock* and *Bmal1* might have a detrimental effect on reproductive health [22,24]. Disruption of a single clock gene, in this case, specifically *Bmal1*, is enough to disrupt the reproductive cycle [24,32,33]. The extent to which other circadian clock genes affect the reproductive cycle is not fully known, however, there is evidence that disruption of these circadian clock genes will cause issues with most aspects of reproduction. As we know, reproduction is made of several processes, including, trigger ovulation, ovulation, fertilization, embryo development, uterine preparation, embryo implantation, placental support of fetus, zygote stage, embryonic stage, and the fetal stage [34,35,36,37,38]. During the past twenty years, circadian clock research has increased each year, and research pertaining to circadian clock regulation of reproduction has also increased (Figure 2). In this focused review, we will first introduce circadian clock proteins and then discuss their known, or hypothesized, roles in reproduction from recent research. 

## 2. Light Cycle

Studies have shown that there is an increased risk to pregnancy in female night-shift workers [39,40,41,42]. Exposure to artificial light at night has caused great concern as it leads to chronodisruption which harms the human biological clock, and causes possible negative effects in human pregnancy [39,43,44,45,46,47,48]. It has been proposed that disrupting the rhythmicity of the circadian system, as shown by an expected anomalous physiological response and gestational chronodisruption, can be translated from mother to offspring [49,50,51]. Studies investigating flight attendants demonstrated that miscarriages have an association with their sleep patterns during work, suggesting, disruptions of circadian rhythm may actuate a miscarriage [52,53,54]. To test the hypotheses surrounding circadian clock genes and reproduction, Goldstein et al. carried out experiments using mice [41]. They found that implantation of the embryo in the uterus is negatively impacted by circadian disruption, only impairing the uterus does not explain the negative pregnancy outcome during the maternal disruption of the circadian cycle. Goldstein et al. pointed out contributors to embryo quality, such as ovarian factors, and early embryo development may be the reproductive mechanisms most vulnerable to the detrimental effects of circadian disruption [41]. Impairment of the uterus by disturbing the mother’s circadian cycle alone is not enough to disrupt the whole pregnancy. Therefore, the hypothesis is that there must also be upstream circadian disruptions [41]. Goldstein et al. postulated that both sets of these circadian genes must exhibit disruption in order for the pregnancy to fail [41]. If these circadian clock genes could be harnessed and redirected, they could be used as a potential cure for female infertility. In addition, repeated shifting of the light/day cycle can reduce the number of pregnancies and implantation sites in animal models [55]. This leads to a dramatic reduction in pregnancy success in mice [56]. This explains the relationship between circadian clock alterations and miscarriages and provides insight into the potential role it upholds within steroidogenesis in the human ovary.

## 3. Embryonic Brain SCN

Many of the body’s processes and physiology function follow a natural circadian rhythm on a 24-hour day-night cycle. However, a female’s fertility can be affected by the amount of light in modern society, shift work, or crossing time zones. Previous research has reported that light/dark cycle, genetic or environmental manipulations that altered the SCN timing signal and disrupted the circadian rhythms of older female mice affected reproductive cycling and function. Mammals are able to adapt to environmental changes, due to crucial biological functions of the SCN, particularly through circadian oscillators. The SCN exhibits rhythms of circadian clock gene expression and matures as the organism grows and develops. Circadian rhythms are synchronized by the SCN. This “master clock” relies on the functional role of the core clock proteins, *Clock* and *Bmal1*, which enables clock gene expression. As mentioned before, these circadian clock genes are all expressed in animal and human placenta, and their expression shows a potential circadian rhythm [26,57]. Several studies have shown the crucial role that the circadian clock and SCN play in female reproductive function [21,22,58]. For example, there have been SCN ablation studies that have revealed the role this central clock plays in regulating estrous cycles and ovulation in female rats [29,59]. The importance of the central clock in reproductive biology is also supported by the observation that deletion of the central clock, which occurs in SCN lesions or global deletion of *Bmal1*, corresponds to defects in estrous cycling and ovulation [60].

The hypothalamic SCN begins to regulate circadian rhythms during days 12–15 of gestation in mouse models [61]. Comparatively, the SCN is discernible as a discrete structure around the 18–30th week of pregnancy in humans. There is expression of *Per2* associated with circadian rhythm at embryonic day 16 (after fertilization), which is approximately equivalent to the end of the first trimester in humans [61]. Not only do these oscillations begin in the absence of vasoactive intestinal polypeptide (VIP), which is located in hypothalamic SCN (plays an important role in many biological functions) VIP-deficient mice exhibit severe disruptions in their ability to express a coherent rhythm in constant conditions. Per2 gene expression increased in intensity as time goes on, up to embryonic day 18 (although not to the adult levels of intensity) [61,62,63]. The *Per2* circadian rhythm can even emerge in mice lacking a circadian clock such as *Bmal1*. Immunohistochemistry study determined expression of *Per2* on day 18, and expression of *Per1* on day 18 to day 19 in SCN [64]. These results suggest that the SCN’s ability to generate circadian oscillations at this age is a property specific to SCN development and differentiation [63]. The development of oscillations in vivo may be due to the tissue’s autonomous programming, which could occur in isolation without signals from elsewhere in the embryo or from the mother. These data also show that the human SCN could be functional at the start of the second trimester (14 weeks of pregnancy) [65]. In order to determine when the fetal SCN clock develops in vivo, and whether a circadian rhythm results from a functional fetal clock, Houdek et al. demonstrated that at embryonic day (E19), rhythms in *Per2* and *Bmal1* expression were absent in the fetal SCN [66]. However, the expression of Rev-erbα (nuclear receptor subfamily 1, group D, member 1, Nr1d1), and other genes related to cellular activity were being driven rhythmically [66]. This suggests that the fetal clock does not operate at E19, but is functional at E21. Houdek et al. speculated that during the early stages in vivo, the developing fetal SCN clock could theoretically be entrained by oscillation of *Nr1d* which may be driven by the maternal, rather than the fetal circadian system [66]. During these experiments, it was found that the rat placentas expressed seven clock genes in a very zone-specific manner [66]. 

It is well known that the relationships between maternal and fetal circadian signals are complex and essential for a successful pregnancy. Mark et al. found that the potential effect of changes in these systems could extend into life, post-birth [34]. To evaluate such variables mentioned above in a rat model, *Bmal1*, *Per2*, *Per3*, *Cry1*, and *Rev-Erb*α mRNA gene expression showed circadian variations since postnatal day 3 (P3), and a reversal of their acrophases from P14 [34]. Additionally, the circadian rhythmicity of *Clock* was only demonstrated at P3, P16, and P21 [34]. This investigation reveals profound significance, as it is unique in addressing the ontogeny of clock genes, which confirms a progressive postnatal maturation of their circadian variation.

It is also important to understand each gene’s role in reproduction. The next section will delve, in-depth, into each known circadian clock gene and their respective roles in reproduction.

## 4. Circadian Gene Involved in Reproduction

### 4.1. PER1 1, 2, and 3

*Per1*, *Per2*, and *Per3* are a class of circadian clock genes, which act as transcriptional repressors, forming a core component of the circadian clock [12]. They are the three members of the period circadian protein homolog. *Per1* is associated with cell proliferation and apoptosis and is involved in the initiation and progression of several cancers such as head-neck carcinoma, prostatic cancer, colorectal cancer, breast cancer, and endometrial cancer [67,68]. *Per2* is also associated with cellular proliferation and differentiation, playing an important role in the development of breast cancer, milk duct, and maintaining the polarity and morphology of the mammary epithelium [67,69,70,71]. *Per3* is associated with poor morning gastric motility and may have a role in the time-keeping function of the gut [72]. *Per3* in knockout (KO) mice has been shown to have a pivotal role in the embryonic development of the cerebral cortex [73].

*Per* mutant females need to have significantly more embryonal implantations in the uterus, in comparison to successfully delivered offspring. Regarding single *Per1* and *Per2* mutants, although fertile, they exhibit lower reproductive success than the control group, as occurs in aged wild-type (WT) mice. The role of *Per3* in regulating metabolism and adiposity has also been described in animal studies [74,75]. *Per1* and *Per2* mutations cause advanced aging [76]. Aging single mutation female *Per1*^−/−^ and *Per2*^−/−^ mice, bear smaller litters than their *WT* counterparts. In addition, aging *Per1^m/m^/Per2^m/m^* double mutation mice have a significantly reduced number of ovarian follicles, displaying a decrease in female fertility starting approximately at 20 weeks and have significantly fewer pups born from 32 weeks old and onwards [77]. The *Per2* oscillation was increased in uteri endometrial stromal cells (UESCs) during implantation, and decreased during differentiation [78]. Cellular differentiation interferes with the circadian clockwork in differentiating cells. The circadian rhythms of *Per2* mRNA and its proteins can be seen in the uteri of pregnant rats during the implantation stage. Conversely, no circadian rhythm of the *Per2* gene was observed in the uteri during differentiation [78]. The circadian rhythm of *Per2* protein also showed a peak around early light periods and a dip around the onset of dark periods. However, the peak time of *Per2* protein level lagged nearly four hours behind its transcript. Recently, Zhang et al. have shown that *Per1* also plays an important role in the regulation of progesterone in female production. Zhang et al. found *Per1* knockout significantly down-regulated the expression of some progesterone receptor target genes and impaired human endometrial stromal cell decidualization via decreased FOXO1 (forkhead box O1) protein level [79]. *Per1* and *Per2* mutants exhibit lower reproductive success than controls, illustrating that proper oscillations of the core clock genes in reproductive tissues are crucial for orderly reproductive function. It remains to be determined whether *Per3* plays a similar role in female reproduction.

### 4.2. CRY1/CRY2

Cryptochrome-1, or *Cry1*, has major importance in the maintenance of circadian rhythmicity [80]. *Cry1* is expressed in most organs, tissues, and cells, and encodes transcription factors that regulate the circadian clock in mammals [81]. *Cry1* can heterodimerize with *Per1*, *Per2,* and *Per3*, to interact with the *CLOCK: BMAL1* complex [80,81]. This serves to suppress the transcription-promoting activity of the complex for genes such as *Per1*, *Per2*, *Per3*, *Cry2*, and *Wee1* (WEE1 G2 Checkpoint Kinase), in cells and different tissues with a ticking circadian clock [81]. Cry1 is known to regulate DNA damage repair, cell proliferation, and several biological processes [32,82,83]. *Cry1* in female mice can stop meiosis of the oocytes and preimplantation of embryos, although that is not involved in circadian clock regulation [84]. Overall, the role of *Cry1* in maintaining normal testis is important for development and function. By contrast, the *Cry2* gene is only shown to be involved in the reproduction of diapausing animals through the seasons. It remains largely unclear; however, what part Cry2 plays in reproduction and more work will have to be done to identity this exact role in the future.

### 4.3. CLOCK

Animal model studies, particularly in the setting of obesity, diabetes, atherosclerosis, or other metabolism syndrome conditions by Clock genes, have led to an increase in recognition that a multitude of rhythmic functions, such as reproductive tissues in mammals, are controlled by molecular clockwork [5,13,15,48,85]. *Clock/Clock* mutant mice have differences in pregnancies, with a higher rate of fetal absorption, serious dystocia, morphological abnormalities, and lower serum progesterone and estradiol levels [24,86]. When considering lactation, *Clock* mutant mice do not have a significant peak of either crouching behavior or prolactin, and the amount of secreted milk is lower than that of wild-type mice [24]. Circadian rhythms implement necessary functions for proper reproduction, sparking interest in evaluating whether there is a link between *Clock* genetic variants, and the impact of idiopathic recurrent spontaneous abortion (IRSA). Hodzic et al. explored groups consisting of participants with IRSA and investigated polymorphic sites in their *Clock* gene of 284 women [87]. Hodzic et al. used single-nucleotide polymorphism selection and genotyping to uncover a correlation between these variables in the genotype distribution of rs6850524 and rs11932595 in the *Clock* gene [87]. Furthermore, evidence demonstrates that the G allele under the dominant model (GG+GC/CC) for rs6850524, as well as the G allele under the dominant model (GG+GA/AA) for rs11932595, may serve as risk factors for recurrent spontaneous abortion (IRSA) [87]. Thus, the findings provide a reason to believe that the variability of the *Clock* gene may be connected with IRSA, prompting a need for further analysis.

Prior studies have indicated that the age-related decrease of melatonin is a consequence of functional changes linked to this system, which controls the sleep/wake cycle. Recently, Semenova et al. showed the circadian rhythms of melatonin secretion in menopausal women and its association with the *Clock* 3111T/C polymorphism with regards to their ethnicity [88,89,90]. The study included 403 menopausal women from both Caucasian and Asian races, who were evaluated based on their diurnal sleepiness (using subthreshold insomnia and Epworth Sleepiness Scale), polymorphic genotyping, and a four-time per day collection of saliva samples for melatonin determination. Higher melatonin levels (1.40 times, *p* < 0.05) were detected in the early morning hours in the carriers of the TT-genotype compared to that in a group of carriers of the minor 3111C- allele [89]. Significant differences in the melatonin levels were found between the control and main group of Asian women carriers of the TT- genotype. This group had a lower hormone level during the day, evening, and night hours in women with insomnia (1.68, 1.80, and 2.13 times, respectively) [89]. It was postulated that in the course of evolution, the allele played a protective role in the development of insomnia [90]. In addition, the Clock gene demonstrated a correlation with the male reproductive system and mutations of the gene have been shown to impact fertility. Ran-binding protein 9 (RANBP9) is a key factor in the development of the gonad [91]. With the intent of investigating spermatogenesis (and the extent to which the functional significance of *Clock* is involved), novel interacting proteins of *Clock* were determined using a yeast two-hybrid assay with cDNA fragments of the *Clock* PAS A domain and human testicular tissue. *RANBP9* may prove to be a novel *Clock*-binding protein and show a direct interactive role between *Clock* and *RANBP9* occurring both in vivo and in vitro [92]. Additionally, both *Clock* and *RANBP9* may be a plausible component of splicing in spermatogenesis [92]. Essentially, *Clock* plays a role in the male reproductive system, and can be related to further studies that seek validation of the relationship between the role of circadian clock genes and spermatogenesis.

Neuronal PAS Domain Protein 2 (NPAS2), is an analog of CLOCK. Like the Clock gene, it has been associated with autism, seasonal variation of sleep length, social activity, mood, weight, appetite, and energy level [93,94]. Kovanen et al. showed, NPAS2 rs2305160 A allele-carriers had lower Global Seasonality Scores a sum score of six items, and carriers of the “A” allele, at NPAS2 rs6725296, had greater loadings on the metabolic factor (weight and appetite) of the global seasonality score from a health interview of an individual living in Finland [94]. This study suggested that NPAS2 gene variants are associated with reproduction. Understanding CLOCK and NPAS2 can lead to improved treatment strategies for reproduction.

### 4.4. BMAL1

Circadian clock genes contribute to reproductive processes in mammals. BMAL1 plays an essential role in female reproduction [60,95]. This includes several phenotypes such as the display of irregular estrous cycles, late onset of puberty, absence of proestrus LH surges, implantation failure, and progesterone-dependent implantation failure, as seen in global *Bmal1****^−/−^*** female mice [60,95]. Similarly, in global *Bmal1^−/−^* females, the deletion of *Bmal1* in Steroidogenic Factor-1 (SF1)-Cre cells leads to implantation failure associated with low progesterone levels [96]. In *SF1-Bmal1^−/−^* female mice, the failure of steroidogenic compartments of the pituitary (i.e., gonadotrophs), adrenal gland, or ovary can be seen. Liu et al. also found that in mice who received progesterone, pregnancy could be sustained, and the females had normal embryos, a normal number of implantation sites, and normal embryo development overall when compared to that in *WT* controls [96]. The circadian rhythm within the ovary is what determines embryo implantation success.

*Bmal1* plays a role in the molecular clock of ovarian steroidogenic cells, the production of progesterone, and other aspects of female reproduction. Studies of this mechanism show that oxidative stress may impair oocyte quality, fertilization, and embryo development, and excess ROS can reduce oocyte quality, fertilization, and embryo development in *Bmal1*^−/−^ female mice [97]. It is possible that in *Bmal1*^−/−^ females, the relatively higher fertilization rate and blastocyst number in vitro were due to the effect of a potent antioxidant contained in the G-series culture mediums that were used in the experiment, while in vivo the oocytes/early embryos were exposed to excess ROS in the oviduct. Xu et al. have tried to explore the effects of the disruption of female circadian rhythm on oocyte fertilization, pre-implantation embryo development, and blastocyst implantation [97]. During natural ovulation, the ovulated oocyte number of *Bmal1*^+/+^ mice was higher than that of *Bmal1*^−/−^ mice. They found significantly lower levels of fertilization and obtained blastocyst numbers in *Bmal1*^−/−^ mice compared with that in *Bmal1*^+/+^ mice, after superovulation and being mated with wild-type males [97]. This study, which is consistent with other recent studies, showed that female *Bmal1*^−/−^ mice were infertile [98]. *Bmal1*^−/−^ mice had lower levels of fertilization, *Bmal1*^−/−^ mice obtained blastocyst numbers compared with that of *WT* mice after superovulation and being mated with *WT* males [98]. Additionally, Mereness et al. reported that the deletion of Bmal1 had locus in ovarian granulosa cells (GCs) (Granulosa Cell Bmal1 KO; GCKO) or theca cells (TCs) (Theca Cell Bmal1 KO; TCKO) [99]. Mereness et al. also found phasic sensitivity to LH (luteinizing hormone) shown in WT littermate control (LC) and GCKO mice but not TCKO mice. TCKO mice were able to alter LH with impaired fertility [99]. TCKO mice were able to alter patterns of LH receptor mRNA abundance in the ovary but with less effect on the reproductive cycles, preovulatory LH secretion, ovarian morphology, and behavior. This indicates the process of follicle development and/or ovulation was at least partly affected due to disruption in circadian rhythm [99]. The theca cells is a pacemaker that modulates phasic sensitivity to LH that contributes to the timing and amplitude of ovulation, indicating an adverse environment existed before ovulation [99]. These data suggest that *Bmal1* likely plays a more important role in reproduction than previously believed.

### 4.5. Nocturnin

Under the control of the circadian clock, *Nocturnin* has rhythmic expression in multiple mouse tissues [11,100]. *Nocturnin* is a hydrolase enzyme that is involved in metabolism and its expression is controlled by the rhythmic circadian clock. It is encoded by the Circadian Deadenylase NOC gene located on chromosome 4 [100,101]. *Nocturnin* not only plays a role in brown adipose tissue, metabolism amplitude, and lipid absorption, but also regulates mice reproduction [17,100,101,102,103]. *Nocturnin* has been shown to be a clock-controlled deadenylase in mouse oocytes and early embryos, although the circadian deadenylase *Nocturnin* expression has not been shown to be rhythmic in preimplantation embryos [101,104]. However, high level of *Nocturnin* expression has severely harmful effects on early embryonic development. Overexpression of *Nocturnin* has shown to significantly increase the expression of fatty acid-binding protein 4 and peroxisome proliferator-activated receptor-γ2 [105]. The level of *Nocturnin* RNA expression has been shown to be the highest in mice oocytes, but it decreases after fertilization, with a slight increase after the 4-cell stage, up to the blastocyst stage [104,105]. *NOCTURNIN* protein expression levels have been shown to be constant during preimplantation in mice. Even after RNA levels decreased, there is evidence to suggest that there is some post transcriptional regulation of the gene’s expression and *Nocturnin* is required for embryo maturation to take place.

## 5. Neural Regulation

In mammals, the SCN clock can work through various neural and endocrine inputs to peripheral tissues to align the sleep-wake cycle with behavioral and physiological oscillations, including body temperature, hormone levels, feeding, and metabolism [106,107]. Neurons have been shown to play an important role in reproduction [108,109]. The effects of the alteration of circadian rhythm via chemical sympathectomy (with 6-hydroxydopamine) or by cutting out a section of the vagus nerve have been shown in pregnant sheep [110]. The results showed a definite and modulating circadian rhythm of sleep cycling in fetuses. However, state-related cardiovascular rhythms were significantly modulated, indicating that the sympathetic nervous system, or vagal activity, is essential for generating cardiovascular diurnal rhythms in the late-gestation fetus [110]. Recently, Padilla et al. showed that *Kiss1^ARH^* neurons (hypothalamic arcuate nucleus Kiss1) affect circadian function [111]. Kisspeptin is an essential neuropeptide for reproduction, with high levels circulating throughout pregnancy [112,113]. Only female *kiss* receptor knockout mice become obese [114]. However, its full role remains unclear. Samples from healthy, full-term, placentas were taken at various time points during the day and tested. This demonstrated a circadian rhythm to placental kisspeptin levels [115]. De Pedro et al. speculated that kisspeptin plays a role in the timing of delivery, perhaps because it acts as a mediator between melatonin and oxytocin molecules, which are also known to play a role [116]. They found a clear increase in kisspeptin expression for morning deliveries over deliveries later at night [116]. This suggests that kisspeptin’s function in reproduction is controlled by the circadian clock.

Agouti-related protein (AgRP) neurons also play an important role in reproduction [117]. Recently, Cedernaes et al. have reported that mice with AgRP-specific ablation of *Bmal1* (ABKO) mice showed disrupted food intake patterns and increased body weight. ABKO mice have shown to increase hepatic gluconeogenesis and altering metabolism, suggesting that the molecular clock plays an important role in AgRP neurons [106]. How is circadian clock regulate neurons sensing this reproduction? It will be interesting to interrogate further whether such neurons sensing of circadian clock occurs in reproduction. Recent studies have shown that the evaluation of chronic AgRP neuron activation on female fertility has been tested using clozapine N-oxide drinking water. This showed a five-day delay in infertility and an estrous cycle delay, together providing support that enhanced AgRP signaling weakens fertility [117]. The results provide a better understanding of the mechanical capabilities of AgRP neurons. It was revealed they attenuated fertility through inhibition of neuroendocrine reproductive-related neurons. Thus, concluding that several neurons participate in reproductive processes.

## 6. Hormone Regulation

### 6.1. Melatonin

SCN neurons synchronize peripheral tissue clocks through not only neuronal, but also hormonal pathways [2,24]. Circadian rhythm and melatonin affect fetal and maternal health and human reproductive success. The use of shift workers and electric lighting has disrupted this cycle and disturbed the optimal levels of melatonin in the blood as well [118,119]. The levels of melatonin in the follicular fluid during ovulation are higher than the levels found in human blood [120,121]. Several studies have reported the role of melatonin is seen not only in the labor and delivery mechanisms but also in ovulation and early pregnancy. Melatonin is produced at several sites in the ovary, and these, along with melatonin from the blood, help regulate the estrous cycle and protect against oxidative stress that contributes to multiple complications in human pregnancy [118,119,122].

When the mother maintains a normal, light/dark, and sleep/wake cycle, it helps to stabilize and maintain the fetus’s circadian clock. Women must maintain an undisturbed light/dark cycle. This helps maintain the circadian clock and preserve the melatonin cycle. Ongoing or extreme disturbances of this rhythm can lead to an adverse effect on the fetus/newborn [122]. Owing to this, it is suggested that the mother, especially during the third trimester, should avoid such disturbances, including shift work and bright light at night [123]. Due to melatonin levels being higher at night and its link with oxytocin, higher melatonin levels may account for higher chances of childbirth at night. 

Whenever this light/dark cycle is disturbed, either because of nighttime disturbance by artificial light or by shift work, it throws off the mother’s circadian clock and suppresses the melatonin cycle, thereby affecting the developing fetus. Melatonin has many effects on the body from inducing a state of sleep to regulating circadian rhythms. Since the embryo cannot produce its own melatonin until after birth, it is reliant on melatonin from the mother [118,122,123]. This maternal influence seems to follow the embryo from oocyte through normal development, until birth. The levels of melatonin increase throughout gestation, first increasing at night after 24 weeks of gestation and then increasing significantly after 32 weeks [118,122]. The addition of melatonin has a positive outcome on high-risk pregnancies. The effects of melatonin may not just be limited to the regulation of circadian rhythms, but further investigation is required. Maternal melatonin is low during the day and increases at night. Additionally, the suppression of melatonin via continuous light exposure had several detrimental effects on fetal growth. It limited intrauterine growth, affected the levels of expression of several clock genes, and lowered the levels of corticosterone, and modified its usage [118,122]. However, these effects were reversed when the mother received a daily injection of melatonin. This is also backed by previous studies that show the negative effects of shift work, jet lag, and daylight savings time on pregnancy and in vitro fertilization (IVF) outcomes [124,125]. This is particularly useful in IVF since a primary cause for infertility is poor oocyte quality. Since it is well established that melatonin helps combat poor oocyte quality and mutations during maturation, melatonin treatment during human pregnancy may help combat some of these stresses and could be used as a treatment for infertility in some cases [125]. Melatonin is already used in some cases during assisted reproductive technologies and IVF. Treating this disruption in pregnant mothers with melatonin can reset the fetal clock via the adrenal gland [125]. Fetuses, whose mothers were exposed to constant light, had lower weight, and the constant light exposure had a negative effect on the fetal circadian clock in the adrenal gland. Maternal exposure to constant light has a negative effect on the cellular response to the adrenocorticotropic release of corticosterone and relative mRNA expression [126]. Fetal growth can be affected in addition to fetal adrenal function due to maternal exposure to constant light [127]. However, these effects could be reversed when the mother receives a daily dose of melatonin during the subjective night.

Recently, Zhang et al. showed the relationship between long light exposure and negative effects on embryo implantation and pregnancy success (mimicking light pollution). Female mice who underwent spontaneous estrous were placed with fertile males and then checked for plugs the next morning [128]. These plugged females were then used for the experiment. The administration of melatonin rescued the negative effect of long light exposure. Melatonin probably increases 17β-estradiol levels during pregnancy and upregulates tumor protein p53 expression by activating melatonin receptors type 1 or 2 in the uterus [128]. This activation likely changes the uterine microenvironment for the better and increases the chance of a positive pregnancy outcome via increased successful embryo implantation. In short, melatonin is important in the development of the fetal circadian clock, helps with the development of the neurological and endocrine systems, and helps protect the embryo/fetus from metabolic stresses that can cause damage to the growing pregnancy.

### 6.2. Estrogen

Estrogen, as an ovarian hormone, is associated with suppressed food intake and produces important anti-obesity and antidiabetic effects in female animals [129,130]. It has shown that circadian clock gene Per2 has a link with the estrogen receptor [131]. Recently, Nakamura et al., showed that estrogen directly affects the timing of the molecular clock in the uterus via an estrogen receptor-mediated response [132]. In addition, estrogen has been shown to differentially regulate the expression of *Per1* and *Per2* genes between central and peripheral clocks and between reproductive and nonreproductive tissues in female animals [132]. Estrogen has been known to account for this sexual dimorphism and is abolished in postmenopausal women [130,133,134]. In addition to its limited role, this hormone may also play another, yet unappreciated, role in the regulation of the circadian clock and reproduction.

### 6.3. Cortisol

Cortisol is a steroid hormone, our body’s main stress hormone. It is one hormone in the glucocorticoid class of hormones, to aid in the metabolism of fat, protein, and carbohydrates [124]. Cortisol also plays an important role in reproduction. In the rodent and human fetus, the diurnal rhythm of cortisol is observed to have the opposite pattern to the maternal rhythm. However, the adrenal circadian rhythm is not synchronized with the clock time after birth [124]. A few months later, a 24-h rhythm can be seen. In a newborn infant, the peak of cortisol level is observed in the late afternoon, in correspondence with the birth time [135]. Iwata et al. suggested that the adrenal circadian clock might play an important role in controlling reproduction [136]. To further determine the role of cortisol in reproduction, cortisol samples were collected at home from the saliva, both at night and first thing in the morning for analysis. There were no group differences in evening or morning cortisol levels. However, children with higher levels of prenatal cocaine exposure showed a blunted increase in cortisol levels between evening and morning measurements, especially compared to non-exposed children [136]. These studies suggest that extensive maternal use of cocaine during pregnancy may constitute constant stress, which results in increased maternal and fetal cortisol secretion and prolonged exposure to elevated cortisol levels. Thus, further research regarding these factors is warranted to know more about the involvement of circadian regulation performed by cortisol in the reproductive process.

## 7. High Fat Diet & Time-Restricted Feeding Regulation

There is strong evidence that high caloric intake has negative impacts on principal body functions, such as endocrine effects, altered liver metabolism, and cholesterol imbalance [137,138,139]. Developmental programming, an action associated with nutrition during pregnancy and early life, imposes continual effects on the health of offspring, as shown through the reproductive repercussions of high-fat diets (HFD) [140,141]. It has been demonstrated that high fat and sugar intake induces alterations in circadian clock function, which seems to be a factor in female reproductive timing, since such changes may harm synchronization between circadian rhythmicity and central or peripheral components. Recently, several studies have shown that high caloric intake leads to premature aging, altered sleep, and disruption in circadian rhythmicity [141,142,143,144]. Highlighting their significance, high-fat diet intake affects the ovarian circadian clock [145]. Circadian core gene expression within ovarian cells revealed changes as a result of maternal and post-weaning high-fat diets [142,145]. Time-restricted feeding (TRF) has been known to prevent body weight gain associated with HFD feeding ad libitum in all genotypes without reducing food intake or increasing activity [146,147,148]. We have shown that TRF modifies clock genes and abetalipoproteinemia genes microsomal triglyceride transfer protein (*MTTP*) expression pattern. The *Mttp* KO mice died early during embryonic development, with reduced lipoprotein secretion in heterozygotes and embryonic lethality in homozygotes [19,149,150].

Several studies have suggested that erratic eating patterns increase the risk of disease. A defined daily feeding–fasting rhythm, as in TRF, is positively related to reducing risk of breast cancer and other chronic diseases [151,152,153]. Prior research has shown that TRF can negatively influence LH pulsatility in prepubertal cycling gilts during ovarian development. Furthermore, TRF has been shown to decrease gonadotropin concentration in humans [154,155]. An early study showed that TRF modulates neuronal orexigenic neuropeptide Y (NPY) gene expression during the phase with low leptin levels and the action of leptin on LH secretion via variation in the availability of glucose. TRF has been known to increase hypothalamic NPY gene expression; this might result from the coordinated action of several factors, such as reducing serum leptin and insulin concentrations. Similar TRF effects have been observed in several studies. Increased NPY levels might inhibit GnRH release during some periods of the feeding cycle [156,157,158,159]. In a recent study, ten-hour TRF was shown to reduce weight, blood pressure, and atherogenic lipids in patients with metabolic syndrome. Like light, TRF coordinates internal biological rhythms with the environment. Gestational circadian rhythm of newborn pups is affected by external cues or via the mother’s own circadian clock [160]. Novakova et al. showed that rat’s pups in a regular light-dark cycle with food restriction did not exhibit any noticeable changes in their circadian clock [161]. However, those animals under constant light with food restriction showed a restoration of circadian rhythm, indicating that when regular external clues are lacking, regular feeding by the animal’s mother may help pups maintain an internal clock [160]. Nevertheless, these studies may help understand the important translation of TRF in reproductive health to provide new therapeutic opportunities to treat shift-work induced reproductive failure and improve the health and well-being of infants.

## 8. Future Directions and Perspectives

Although our and other reviews are starting to piece together the early events and identify proteins involved in animal reproduction, very little is known about circadian rhythm disruption in humans. It is well known that animal models, although useful, do not completely mirror the human reproductive system, especially with respect to hormonal fluctuations as well as circadian rhythm disruption. Therefore, the need for more studies on the effect of the disruption of circadian rhythms in human fertility is required. It should be noted, however, that external effects cannot be ruled out, such as the stress of vaginal delivery, including the increase in stress hormones known to affect gene expression. Only two of the genes studied—*Clock* and *Bmal1*, showed any circadian variation [26].

The developing fetus receives essential nutrients based on the mother’s metabolic changes during two specific maternal metabolic phases, the anabolic and catabolic phases. This process of metabolic homeostasis is increasingly recognized to share a link with circadian variation. This is stimulated by the work of clock genes, and also shares importance with maternal carbohydrate and fat metabolism, since glucose and lipids are the fetus’s primary energy source. For example, in mice, a mother’s adaptation to pregnancy includes shifts in clock genes within the liver that results in a reduction of the circadian clock that regulates glucose. This variation in circadian regulation of glucose metabolism, midway through gestation, ensures a sustainable supply of glucose to meet the demands of fetal growth. Investigating the relationship between the internal circadian clock and metabolic abnormalities in pregnancies, serum triglycerides in mice increase in later pregnancy and fluctuate throughout the light/dark cycle. In humans, postprandial serum triglyceride levels are high [98]. These data suggest that there is an increase in serum triglyceride levels in the third trimester after a high-calorie lunch compared to levels in non-pregnant women.

Recently, we have shown that plasma triglyceride showed diurnal rhythm in *WT* mice, and *ApoAIV* (Apolipoprotein A IV) and *MTTP* plays an important role in adult mice hepatocytes triglyceride and Very Low-Density Lipoproteins (VLDL) production. TRF in day time, shift the peak of hepatocytes *ApoAIV* and *MTTP* gene expression in mice with 12 light/12 dark cycle [19,162,163,164]. In addition, Clock proteins play an important role in the diurnal rhythm of plasma triglycerides [19,149]. It will be interesting to interrogate further whether such lipid metabolism genes occur in localized regions from the egg, to the embryo, to the fetus, undergoing triglyceride production and through a similar mechanism as suggested for adult circadian clock regulation. However, variations in the expression of circadian clock genes in different tissues and TRF are the components of maternal metabolic adaptation in pregnancy. Such changes promote variation in circadian expression of metabolic genes involved in glucose and lipid homeostasis. In addition, the nature of the relationship between a mother’s metabolism of carbohydrates, fat, and protein, and energy consumption by the developing fetus remains unclear. Identification of other proteins involved in the reproductive process may provide novel targets for the treatment of circadian clock (sleep) disorder-associated diseases such as reproductive failure.

## Abbreviations

*ApoAIV:*	Apolipoprotein A IV;
AgRP:	Agouti-related protein;
Arntl:	Aryl hydrocarbon receptor nuclear translocator-like protein 1, BMAL1;
ABKO:	AgRP-specific ablation of *Bmal1*;
Clock:	Circadian Locomotor Output Cycles Kaput;
cDNA:	complementary DNA;
*Cry 1/2:*	Cryptochrome Circadian Regulator 1/2;
E:	embryonic day;
FOXO1:	forkhead box O1;
GCs:	granulosa cells;
GCKO:	Granulosa Cell Bmal1 KO;
GnRH:	Gonadotropin-releasing hormone;
IRSA:	recurrent spontaneous abortion;
KO:	Knock out, −/−;
*Kiss1^ARH^*:	Metastasis-suppressor KiSS-1 arcuate hypothalamus;
LC:	littermate control;
LH:	Luteinizing hormone;
NPAS2:	Neuronal PAS Domain Protein 2; m/m: mutant/mutant;
MTTP:	microsomal triglyceride transfer protein;
NPY:	neuropeptide Y;
*Per 1/2/3:*	*Period* Genes Period Circadian Regulator1/2/3;
Postnatal day 3:	(P3); RANBP9: Ran-binding protein 9;
Rev-erbα:	nuclear receptor subfamily 1, group D, member 1, Nr1d1;
SCN:	suprachiasmatic nucleus;
SF1:	Steroidogenic Factor-1;
TCs:	theca cells;
TCKO:	Theca Cell Bmal1 KO;
TRF:	Time-restricted feeding;
UESCs:	uterus endometrial stromal cells;
Wee l:	WEE1 G2 Checkpoint Kinase.
